# Extensive Surgical Emphysema in a Child after Primary Closure of Tracheocutaneous Fistula

**DOI:** 10.1155/2020/3714718

**Published:** 2020-01-30

**Authors:** R. Gurung, B. M. Shakya, H. Dutta

**Affiliations:** ^1^Department of Anaesthesiology, Maharajgunj Medical Campus, Kathmandu, Nepal; ^2^Department of ENT, Maharajgunj Medical Campus, Kathmandu, Nepal

## Abstract

A 4-year-old child had closure of tracheocutaneous fistula under general anaesthesia. He developed extensive surgical emphysema over the face, chest, and upper abdomen immediately in the recovery room. We gave him oxygen supplementation, removed surgical stitch, and inserted a 4 mm tracheostomy tube to secure airway. Chest X-ray ruled out pneumothorax or pneumomediastinum. After a week, a tight bandage was applied which approximated the tissue and helped in the closure of stoma; no suture was applied. The patient was discharged home on the fourth postoperative day. The patient needs close observation in the postoperative period with likely complication in mind. Recognizing early signs and symptoms of respiratory distress with quick intervention is lifesaving during the complication of tracheocutaneous fistula surgery. In absence of pneumothorax or pneumomediastinum, extensive surgical emphysema occurring during primary closure of tracheocutaneous fistula can be treated without inserting any drainage tube.

## 1. Introduction

Surgical treatment of tracheocutaneous fistula (TCF) is the excision of fistula followed by either primary closure or secondary healing. The primary closure provides immediate resolution of the fistula but is associated with complications among which surgical emphysema with massive extension is rare [1], but it may lead to airway obstruction, which can be life-threatening.

## 2. Case History

**Figure 1:**
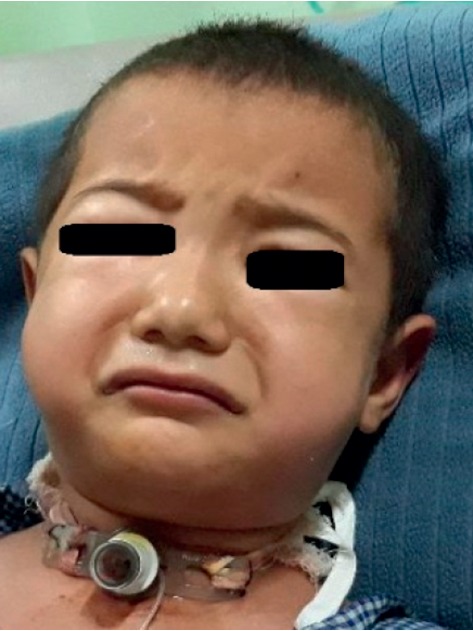
Immediately after operation puffiness of the eyelid, face, and chest.

**Figure 2:**
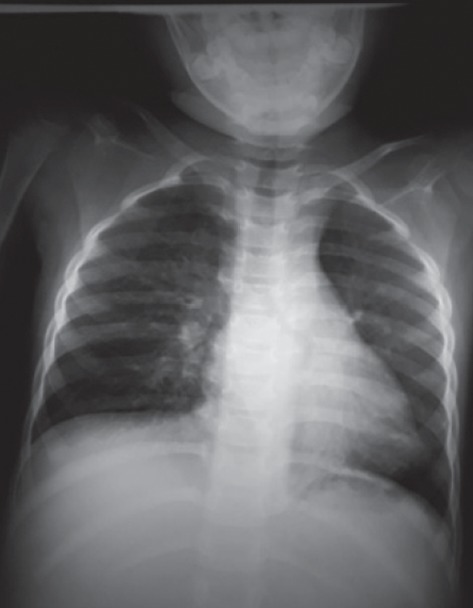
Preoperative chest X-ray.

**Figure 3:**
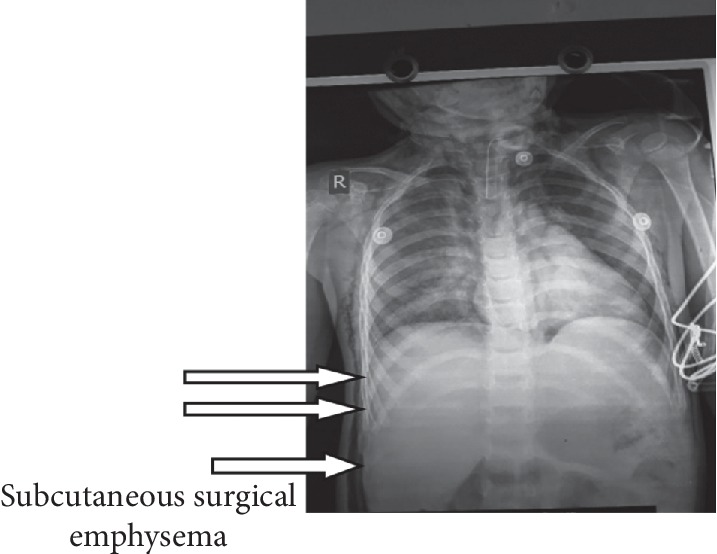
Postoperative chest X-ray.

A 4-year-old child with diagnosis of glottic web had tracheostomy at 15 months of age. At 3 year of age, the metallic tracheostomy tube was removed but tracheostomy opening persisted. Surgeons had scheduled the patient for laryngotracheal evaluation and closure of tracheostomy stoma. Preoperative investigation was within the normal limit. Surgical decanulation done under general anaesthesia was uneventful intraoperatively. Incision closed in two layers; inner subcutaneous tissue sutured with 3′o vicryl and skin sutured with 4′o ethylene. The patient was extubated and transferred to the recovery room. After about an hour, the patient developed respiratory distress and swelling of the eyelid and face. Saturation was dropped to 81% in room air and the heart rate was increased to 140/min. The child had developed rapidly spreading subcutaneous emphysema. Immediately, the patient rushed to the operation theatre and oxygen was supplemented with a facemask. Surgical stitch applied for tracheostomy closure was removed and the tracheostomy tube (size 4 mm ID) reinserted immediately. The capnograph confirmed the correct position and ventilation done through the tracheostomy tube. Saturation gradually increased up to 95%. On pressing, crepitus was present bilaterally over the face, neck, and anterior chest wall and even up to the upper abdomen (Figure 1). On auscultation of the chest, the air entry was bilaterally present but in decreased intensity and there were no rhonchi. The patient was tachycardic. The patient was in the recovery room with continuous monitoring and oxygen supplementation. Chest X-ray showed air collection at subcutaneous tissue of the chest and abdomen but no evidence of pneumothorax or pneumomediastinum (Figures 2 and 3).

**Figure 4:**
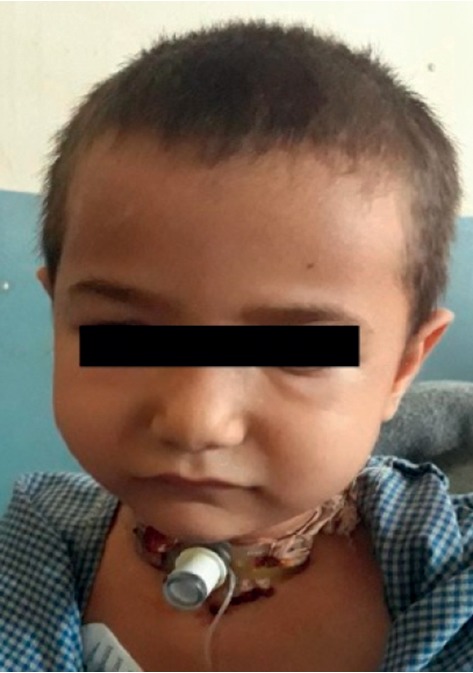
On the 1^st^ postoperative day.

**Figure 5:**
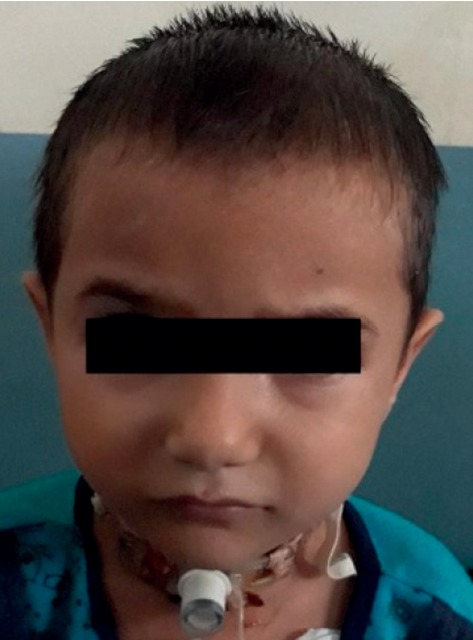
On the 2^nd^ postoperative day.

In the postoperative ward, the patient was closely observed. On the first postoperative day, crepitus over the abdomen and chest was decreased and puffiness of the face was also decreased (Figure 4). The vital was stable on the first postoperative day. On the second postoperative day, crepitus over the abdomen, chest, and face was further decreased (Figure 5). The patient was comfortable on the tracheostomy tube. After a week, the subcutaneous emphysema was completely resolved. Decanulation was done, and the stoma was approximated with bandage but no suture was applied. Postoperatively, there was no complication of airway and the patient was discharged home on the fourth postoperative day. The stoma site completely closed within one month.

## 3. Discussion

Generally, tracheostomy opening closes spontaneously, but sometimes it persists as a fistula. TCF closure is done by excising the fistula surgically and closing the stoma by primary closure or by secondary closure. Between two methods of closure, there is no statistical difference in terms of complication or its outcome [2]. Decision about the appropriate method is made on the individual patient and surgeon [3]. In our case, after first attempt of closure, subcutaneous emphysema developed, which may be because of tight stitching of skin. It caused entrapment of air expired through the tracheal stoma in the subcutaneous tissue. Removal of surgical stitches released the outlet, and thus subcutaneous emphysema had decreased. During the second attempt of closure, skin and subcutaneous tissue were just approximate with tape bandage without any suture. Air did not entrap, and the wound healed overtime with sealing of the tracheostomy stoma.

Complications seen with tracheocutaneous fistula surgeries are like subcutaneous emphysema, pneumomediastinum, pneumothorax, bleeding, respiratory distress, wound infection, and readmission. Incidence of these complications was about 20% [4]. In some tracheocutaneous fistula closure cases, extensive subcutaneous emphysema develops resulting in acute respiratory distress immediately after transfer to the recovery room [5, 6]. Therefore, after each tracheocutaneous fistula surgery, close monitoring of the patient is necessary. The medical personnel should have a vigilant eye over likely complications. They should be able to quickly identify early signs of complications and intervene timely. One of the methods adopted is placing the drain during closure of wound. It may decrease the severity but does not eliminate the complications [5]. That is why keeping the patient under observation with early intervention is a lifesaving procedure.

For any postoperative respiratory distress, we should look for surgical emphysema and its extension. In children, if there is no underlying lung pathology like lung bulla/fibrosis/cavity, then the first step is giving oxygen supplementation as soon as possible. The second step is the removal of surgical stitch, which prevents the further extension of emphysema. Then, airway is secure by inserting the tracheostomy tube. Radiological investigation is performed to rule out pneumothorax, pneumomediastinum, and extension of surgical emphysema.

In children, after primary repair of persistent tracheocutaneous fistula, positive airway pressure ventilation increases the risk of serious respiratory complications. Smith et al. had done a study in 108 paediatric patients who had undergone for tracheocutaneous fistula surgery. The frequency of all postoperative complications was significantly higher in patients administered positive airway pressure ventilation versus those who were not (50.0% vs. 16.7%, *P*=0.015), as were the rates of subcutaneous emphysema, pneumomediastinum, and/or pneumothorax (33.3% vs. 4.2%, *P*=0.005* *). Therefore, we advise to avoid bag mask ventilation and be mindful when using CPAP or BiPAP postoperatively [4].

## 4. Conclusion

During surgical decanulation, skin and subcutaneous tissue should be just approximate to seal the tracheal stoma. Tight suturing of tissue may prevent the release of air and develop life-threatening respiratory distress.

Removal of surgical stitches, securing airway, supplementation of oxygen, and close monitoring of patient are required in the postoperative period for a paediatric patient who had developed extensive surgical emphysema after closure of tracheocutaneous fistula. The patient may not need any surgical nick or insertion of the drainage tube.
